# Real-time fully automated dosimetric computation for CT images in the clinical workflow: A feasibility study

**DOI:** 10.3389/fonc.2022.798460

**Published:** 2022-08-11

**Authors:** Massimiliano Porzio, Choirul Anam

**Affiliations:** ^1^ Department of Fisica Sanitaria, Azienda Sanitaria Locale Cuneo1 (ASL CN1), Cuneo, Italy; ^2^ Department of Physics, Faculty of Sciences and Mathematics, Diponegoro University, Semarang, Indonesia

**Keywords:** automatic, software, water-equivalent, SSDE, CT, dose, real-time

## Abstract

**Background:**

Currently, the volume computed tomography dose index (CTDI_vol_), the most-used quantity to express the output dose of a computed tomography (CT) patient’s dose, is not related to the real size and attenuation properties of each patient. The size-specific dose estimates (SSDE), based on the water-equivalent diameter (*D*_W_) overcome those issues. The proposed methods found in the literature do not allow real-time computation of *D*_W_ and SSDE.

**Purpose:**

This study aims to develop a software to compute *D*_W_ and SSDE in a real-time clinical workflow.

**Method:**

In total, 430 CT studies and scans of a water-filled funnel phantom were used to compute accuracy and evaluate the times required to compute the *D*_W_ and SSDE. Two one-sided tests (TOST) equivalence test, Bland–Altman analysis, and bootstrap-based confidence interval estimations were used to evaluate the differences between actual diameter and *D*_W_ computed automatically and between *D*_W_ computed automatically and manually.

**Results:**

The mean difference between the *D*_W_ computed automatically and the actual water diameter for each slice is −0.027% with a TOST confidence interval equal to [−0.087%, 0.033%]. Bland–Altman bias is −0.009% [−0.016%, −0.001%] with lower limits of agreement (LoA) equal to −0.0010 [−0.094%, −0.068%] and upper LoA equal to 0.064% [0.051%, 0.077%]. The mean difference between *D*_W_ computed automatically and manually is −0.014% with a TOST confidence interval equal to [−0.056%, 0.028%] on phantom and 0.41% with a TOST confidence interval equal to [0.358%, 0.462%] on real patients. The mean time to process a single image is 13.99 ms [13.69 ms, 14.30 ms], and the mean time to process an entire study is 11.5 s [10.62 s, 12.63 s].

**Conclusion:**

The system shows that it is possible to have highly accurate *D*_W_ and SSDE in almost real-time without affecting the clinical workflow of CT examinations.

## Introduction

A computed tomography (CT) scanner is often used to accurately diagnose cancer. In addition, CT is used in the treatment planning of radiotherapy (1). A CT image accurately provides the shape and position of cancer and surrounding healthy tissues, along with organs at risk. CT provides a map of the electron density information for the various tissues to accurately and precisely calculate the dose delivered to the patients during radiotherapy. However, CT employs ionizing radiation, and it potentially induces new cancer in the future (2–6). CT delivers higher ionizing radiation doses than common radiographic examinations (7).

Currently, the quantities used to express CT dose are the volume computed tomography dose index (CTDI_vol_) and the dose-length product (DLP; the CTDI_vol_ multiplied by the scan length) (8). Those values do not directly estimate the patient dose but are measured from the output of the X-ray tube on cylindrical plastic phantoms representing an average patient head (16 cm in diameter and 15 cm thick) and/or torso (32 cm in diameter and 15 cm thick).

Those phantoms do not represent the actual patient dimensions since the dose received by the patient varies according to the size and attenuation of the patient. A small patient would receive a higher radiation dose than a bigger one, even if their CTDI_vol_ is the same (9, 10). To overcome those issues, the American Association of Physicists in Medicine (AAPM) in 2011 introduced a new metric called the size-specific dose estimates (SSDE) (11).

The computation of SSDE relies on the water-equivalent diameter (*D*_W_), a quantity that accounts for patient composition and attenuation properties (12). It is possible to compute *D*_W_ for every CT image using manual (13) or automated techniques (14, 15).

Many studies (16–18) proposed fully automated methods. Özsoykal et al. (19) proposed an automated patient contour after the exclusion of irrelevant objects such as clothes and the CT table from the image. The threshold value was determined through trial and error until a complete successful segmentation of the body contour was obtained. Anam et al. (14) also proposed an automated approach to calculate *D*_W_ in human anatomic regions and phantoms using a region of interest (ROI) that is automatically fitted to the patient border. The automated approach produced an excellent correlation with the manual one (*R* ^2^ = 0.999). Gharbi et al. (20) also successfully proposed an automated approach to measure *D*_W_ based on the Fuzzy C-means classification and edge detection algorithms. Juszczyk et al. (21) proposed an automated segmentation approach to calculate *D*_W_ using a convolutional neural network (CNN) and reported that the proposed method produces accurate results. However, they are retrospective techniques and do not allow for real-time assessment of *D*_W_ and SSDE. Real-time measurements could be useful for assessing whether the correct technique is being applied to the patient, or whether it is necessary for some changes to account for the patient’s size [e.g., tube current modulation (TCM)].

Furthermore, a rapid SSDE computational system would benefit many oncological patients who undergo numerous CT exams and need their exposures tailored to their specific sizes (22) and reduced as low as possible to reduce the risk of relapse and/or radiation-induced new cancer (2–4).

The aim of this study is to develop a fully automated method of *D*_W_ and SSDE measurement for real-time and to investigate the feasibility of its implementation in a clinical workflow, evaluating its accuracy and the time required to do it for a complete CT study.

## Method

### Main algorithm description

**Figure 1:**
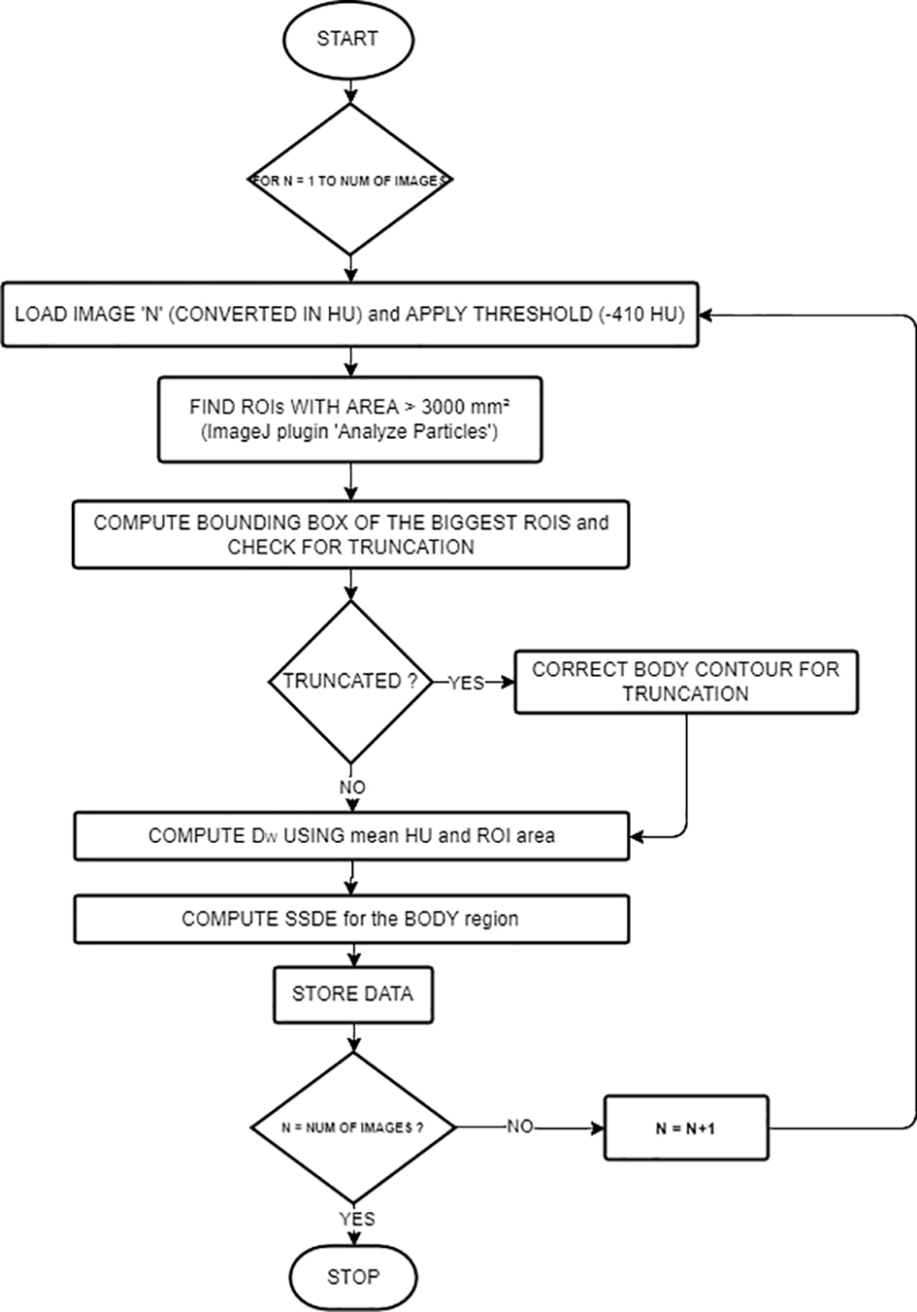
Workflow of the main algorithm for *D*_W_ and SSDE computations.

A previously published work was the basis of the algorithm (14). The workflow is described in **Figure 1**. The language used was Java, and the main third-party library is ImageJ (RRID: SCR_003070) (23). The input was the path on the filesystem of a series of CT images and the path of the text file where the user wants to save the computed dosimetric quantities.

Images were first ordered by the image number read in the Digital Imaging and Communications in Medicine (DICOM) header attribute. The middle slice^1^ then becomes the reference for other study-related information read from the DICOM header (i.e., accession number, station name, series number, pixel dimensions, CTDI_vol_, protocol name, sex, and age). We read the anatomical region from the protocol name attribute.

#### *D*_W_ and SSDE computations

The algorithm implemented a combination of basic segmentation techniques and specific information about the border of the patient’s body using the “Analyze particles” (24) plugin.

For every image, we computed *D*_W_ and SSDE using the following steps: *First*, reading the image—ImageJ opens the images and converts them into Hounsfield units (HU). *Second*, applying the threshold (−410 HU, using the Analyze particles [24) plugin)] in order to detect border regions of interest (ROIs) with an area greater than 3,000 mm². We selected this value (−410 HU) because the border between the patient and its surroundings is skin, with pixel values of approximately 0 HU, while the surroundings outside the patient are air or other materials with pixel values lower than −410 HU. The thresholding produces binary images. However, thresholding alone could not contour the patient completely because of the presence of other objects inside the patient with pixel values lower than −410 HU. To overcome this problem, the plugin implemented edge detection to identify these objects and label them using their areas. We considered the largest area identified to be the border of the patient or phantom. If the plugin finds multiple ROIs, we choose the ROI with the centroid nearest to the center of the image.

**Figure 2:**
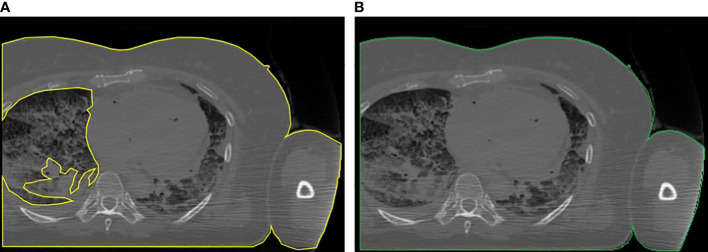
Example of the correction applied to truncated images: **(A)** body region not properly located and **(B)** body region after the correction.

*Third*, computing the bounding rectangle of the ROI to see if the patient’s body is truncated as in (25). If so, we first applied a correction to the ROI for letting the border of the patient not follow the lung contour but go straight along the image sides (see **Figure 2**), and then we applied the correction presented in (25), based on the percentage of truncation of the patient’s body (25) to estimate more correctly the water-equivalent diameter, based on the anatomical region we previously found.

*Fourth*, computing means HU, ROI area, and then *D*_W_ using:


(1) $$D_{W}=2\sqrt{\left[\frac{1}{1,000}\bar{HU{\left(x,y\right)}_{ROI}}\right]\frac{A_{ROI}}{\pi }}$$

*Fifth*, SSDE was computed according to the anatomical region (head or “other”) and the reconstruction diameter. The SSDE for head CTs was computed with


(2) $$SSDE=1.9852e^{-0.0486D_{W}}$$

The SSDE for body CTs, based on the reconstructed diameter previously read from the DICOM header along with the anatomical region inferred from the protocol name, was computed with:


(3) $$SSDE=\{\begin{array}{c} 1.877e^{-0.039D_{W}}\;\text{if}\;\text{reconstr}.\;\;\text{diam}.\leq 320\;\text{mm}\\ 3.7055e^{-0.037D_{W}}\text{if}\;320\;\text{mm}<\text{reconstr}.\;\;\text{diam}.\leq 400\;\text{mm} \end{array}$$

Equation (3) separates CT images with CTDI_vol_ computed for a 16-cm diameter and those with CTDI_vol_ for a 32-cm phantom.

For every image, we also computed the time elapsed from the reading of the DICOM file to the end of computations of all the quantities.

We wrote on a text file the accession number, series number, protocol name, station name, *D*_W_, SSDE, CTDI_vol_, the percentage of truncation—computed as in (25)—and the time elapsed for analyzing the image.

### Real-time software architecture

**Figure 3:**
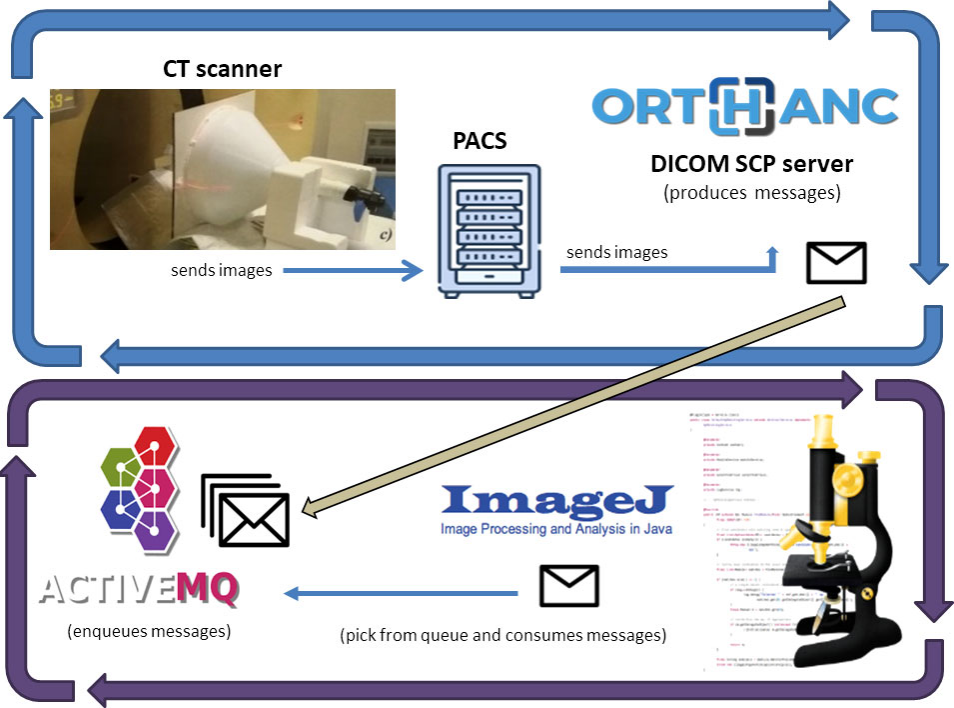
Software architecture of our system: Images received from the Institutional PACS archive are sent for storage to an Orthanc DICOM server. The server sends messages on an ActiveMQ queue using the Lua script. A Java software built on ImageJ API does *D*_W_ and SSDE computations for every message it reads from the queue.

The described algorithm was at the core of the system. We built a software architecture that runs this algorithm without affecting the communication between CT scanners and the Picture Archive and Communication System (PACS). **Figure 3** shows the architecture of our system.

We decoupled the dosimetric computation from the clinical workflow, using a routing rule of the institutional PACS archive. It sent every received CT series to another PACS installed in our Medical Physics Department (Orthanc DICOM storage server (26)).

The Orthanc server uses Lua (27) scripts to monitor DICOM instances and react accordingly. A Lua script filtered the CT series and, if the related metadata (read from the DICOM header) were suitable for the study, then it saved the images on disk and writes a message to an ActiveMQ queue. The message contains the path where the Lua script saved the CT images of each series.

ActiveMQ is an open-source message broker (28) for managing messages received from other software components and letting other components react to each message’s arrival.

Another Java software component is listening for messages on the queue and, for every message, it takes the path of CT images and runs the main algorithm, saving dosimetric information on a text file.

### Algorithm validation

**Figure 4:**
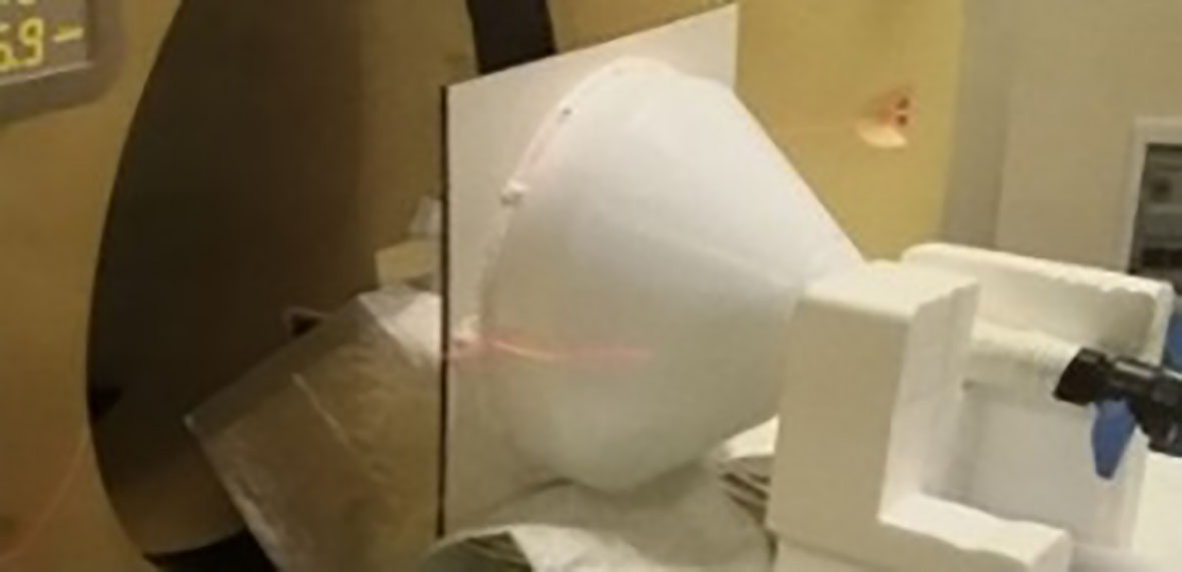
Our funnel phantom (plastic filled with water).

We validated our algorithm using a homemade funnel phantom used in a previous study (29) filled with water (**Figure 4**). It was composed of plastic, and its diameter range was 10 ÷ 34 cm with an effective length of 14 cm (total length: 36 cm). We scanned the phantom on a Philips Brilliance 40 CT scanner and computed the manual *D*_W_ for a very thin slice, to compare automatic *D*_W_ computation with the actual diameter and with *D*_W_ computed manually. The manual computation involved body contouring and collecting statistics (mean CT number and area of the contoured ROI). We repeated the manual calculation on five actual patient series and compared the *D*_W_ to the automatic computation.

### Data collection

We collected data for a week on CT scans of patients with an age of >18 years and with scanning protocols related to head, head and neck, thorax, abdomen, and/or pelvis anatomical regions. We computed *D*_W_ and SSDE from five CT scanners (see **Table 1**). The number of CT series, after the removal of outliers^2^ in the elapsed time, was 1,789 (1,013 abdomen/pelvis, 432 head, and 344 thorax). Summing the elapsed time for images and series with the same accession number, we gathered the time to process a whole CT study. The number of slices collected ranged from 14 to 4,460, with a median value of 585 slices per patient. The total number of CT studies was 430.

**Table 1:** CT scanners used in this study.

Manufacturer	Model
Philips	Brilliance CT 6-slice
Philips	Brilliance CT 16-slice
Philips	Brilliance CT 16-slice
Philips	Brilliance CT 40-slice
Toshiba	Aquilion 64-slice

To show the potential of our approach, we draw the SSDE and CTDI_vol_ vs. slice number for one patient, as well as the *D*_W_ vs. slice number, using the collected data, in order to show the type of information a final user can get with little further effort.

### Statistical analysis

The R language (30) was used to perform all the statistical analysis. We evaluated the differences between the actual diameter and *D*_W_ computed automatically for the funnel phantom with two one-sided test (TOST) (31) (equivalence bounds: ± 7%) and Bland–Altman analysis (32). We also evaluated the difference between manual and automatic *D*_W_ computation using TOST (equivalence bounds: ± 10% for the funnel phantom). On five patient series, we compared automatic and manual *D*_W_ using TOST with 12% as equivalence bounds. We chose equivalence bounds based on the International Electrotechnical Commission (IEC) norm IEC 62985:2019 (Methods for calculating size-specific dose estimates (SSDE) for computed tomography).

We analyzed patients’ data in terms of mean processing time for a single image and for a single study. We computed confidence intervals (CI) for mean times with non-parametric bootstrap using the “nptest” package (33). We set the significance level to *α* = 0.01 for TOST analysis and to *α* = 0.05 for the Bland–Altman confidence interval significance level. All CIs are reported in square brackets, i.e., [lower CI limit, upper CI limit].

Finally, we computed the mean percentage of truncation, following (25), along with its bootstrapped CI using the “nptest” package (33).

## Results

### *D*_W_ of water-filled funnel phantom

**Figure 5:**
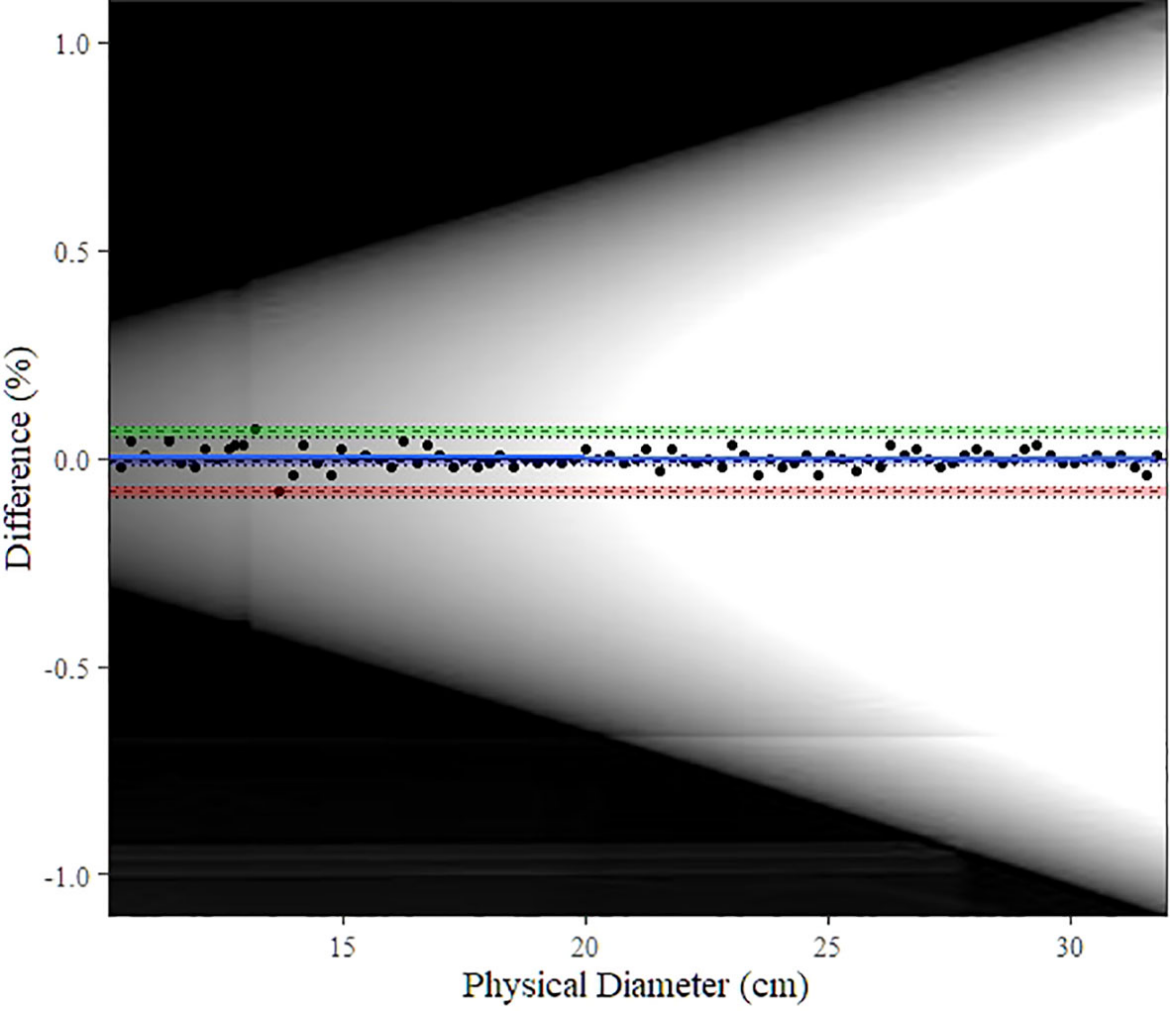
Bland–Altman plot for automatic *D*_W_ computation vs. physical (actual) diameter. The blue line shows the bias estimate with confidence interval (CI) as a blue band. Upper and lower limits of agreement (LoA) are depicted as dashed lines with colored bands as CI (upper LoA**’**s CI: green, lower LoA**’**s CI: red). The background image is the scout image of our water-filled funnel phantom.

**Figure 6:**
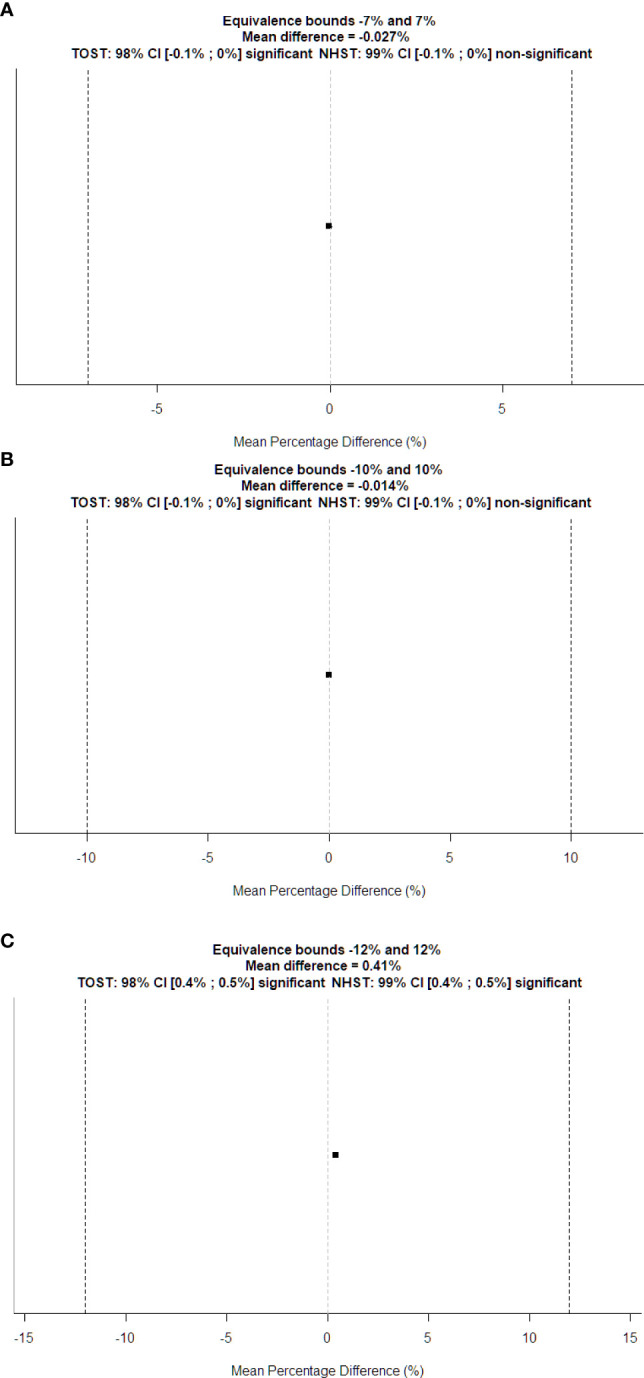
TOST plots for equivalence tests between automatically computed *D*_W_ vs. **(A)** physical diameter and **(B)** manually computed *D*_W_ of the water-filled funnel phantom; **(C)** manually computed *D*_W_ of the patient’s CT series. Dotted vertical lines represent the boundary of equivalence. The squared dot is the mean percentage difference, and the small line through it shows the confidence interval for the standard *t*-test.

**Figure 5** shows the Bland–Altman plot for the *D*_W_ of the water-filled funnel computed and the actual funnel diameter. Bland–Altman bias is −0.009% [−0.016%, −0.001%] with lower limits of agreement (LoA) equal to −0.0010 [−0.094%, −0.068%] and upper LoA equal to 0.064% [0.051%, 0.077%]. **Figure 6A** displays the TOST plot of the *D*_W_ of the water-filled funnel computed automatically and the actual funnel diameter. The *D*_W_ of the water-filled funnel is computed automatically, and the actual funnel diameter is statistically not different from zero (*p* < 0.001) and statistically equivalent to zero (*p* < 0.001), given the ±7% equivalence bounds. The mean difference between the *D*_W_ computed automatically and the actual funnel diameter for each slice is −0.027% with a TOST confidence interval equal to [−0.087%, 0.033%].

**Figure 6B** displays the TOST plot of the *D*_W_ of the water-filled funnel computed automatically and manually. The *D*_W_ of the water-filled funnel computed automatically and manually is statistically not different from zero (*p* < 0.001) and statistically equivalent to zero (*p* < 0.001), given the ±10% equivalence bounds. The mean difference between the *D*_W_ of the water-filled funnel computed automatically and manually is −0.014% with a TOST confidence interval equal to [−0.056%, 0.028%].

### *D*_W_ of patients

**Figure 6C** shows the TOST plot of the *D*_W_ of patients computed automatically and manually. The *D*_W_ of patients computed automatically and manually is statistically different from zero (*p* < 0.001) with a mean difference equal to 0.41% and statistically equivalent to zero (*p* < 0.001) given the ±12% equivalence bounds with a TOST confidence interval equal to [0.358%, 0.462%].

The mean percentage of truncation is 3.58% [3.27%, 3.91%]. Those (small) percentages show that the field of view encompassed nearly the whole-body contour.

**Figure 7:**
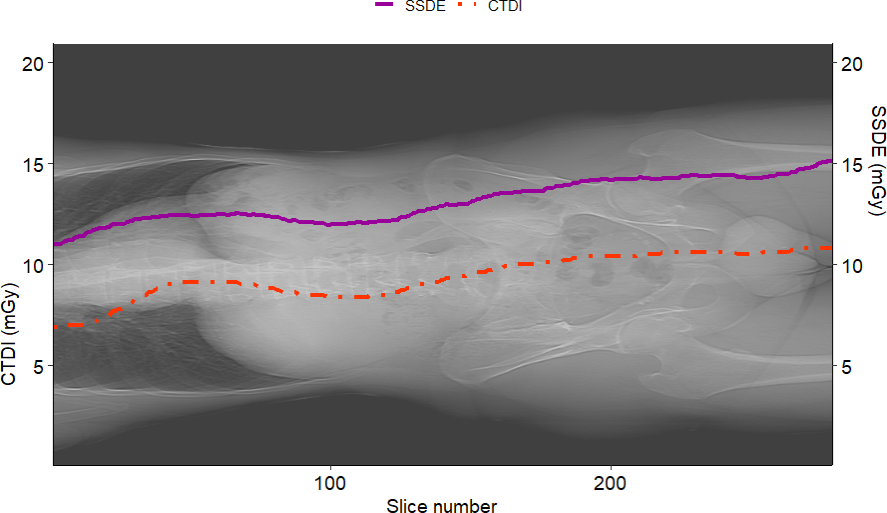
Example of SSDE and CTDI_vol_ plot vs. slice number for a sample patient. The solid curve represents the SSDE computed for each slice, and the dotted one is the CTDI_vol_ read from the DICOM header of the slices. It is worth noting that SSDE is different from CTDI_vol_.

**Figure 8:**
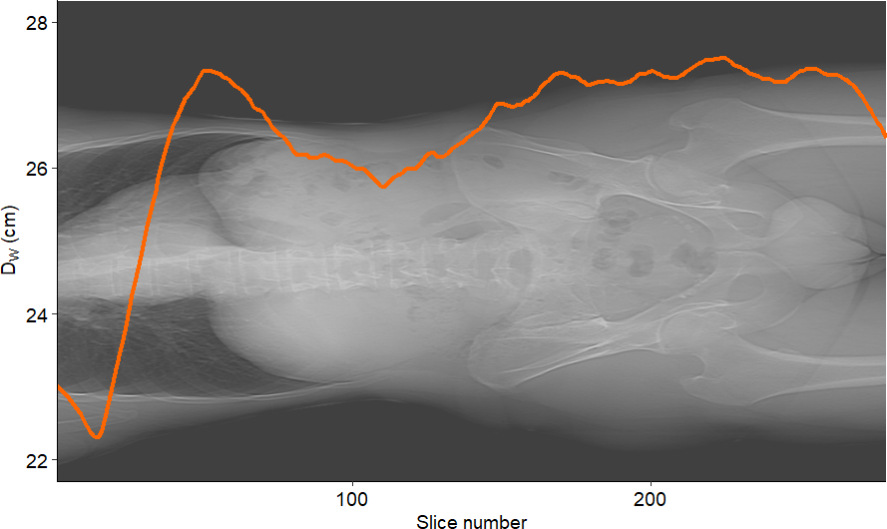
Example of *D*_W_ vs. slice number for a sample patient. The curve represents the water-equivalent diameter *D*_W_ computed for each slice.

**Figure 7** shows the graphs of SSDE and CTDI_vol_ vs. slice number. **Figure 8** displays the plot of *D*_W_ vs. slice number. We created these plots using the package “ggplot2” (34). This type of graph shows the values of *D*_W_ and/or SSDE and CTDI along the slice number, i.e., showing the values along the patient’s anatomy.

### Time for processing

**Figure 9:**
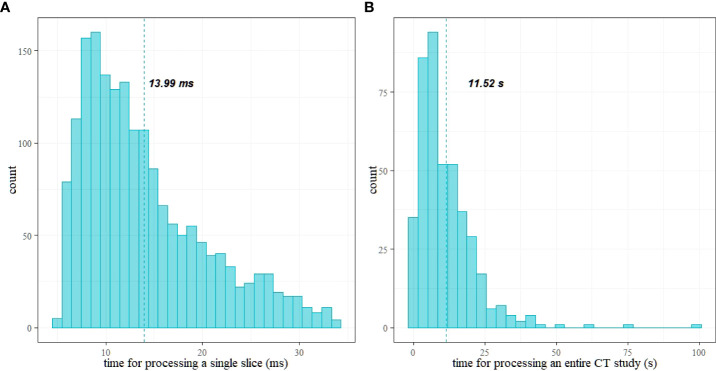
Histograms of time for processing: **(A)** a single CT slice and **(B)** a patient study; vertical lines show the mean values.

**Figure 9** shows histograms of time for processing of a single CT slice and a patient study. The mean time to process a single image is 13.99 ms [13.69 ms, 14.30 ms], and the mean time to process an entire study is 11.5 s [10.62 s, 12.63 s]. **Figure 9** shows histograms of the times elapsed during the processing. **Table 2** reports the summary of the distribution of times to process a whole CT study.

**Table 2:** Summary of time (in seconds) required to process a whole CT study.

Min.	1st Quartile	Median	Mean	3rd Quartile	Max
0.16	4.57	8.53	11.52	15.56	99.2

## Discussion

We developed a software for real-time computation of size-specific dosimetric quantities: *D*_W_ and SSDE. We also evaluated the accuracy of the computation and the usability in a clinical workflow, in terms of time for processing single images and a whole patient study.

The mean difference in the *D*_W_ of the water-filled funnel phantom between the automatically computed and the actual diameter of the funnel phantom is small (−0.027%), as well as the difference between automatically and manually computed *D*_W_ on that phantom (−0.014%). Furthermore, the mean difference between the automatic computation of the *D*_W_ and the manually computed value of a patient’s data is also small (0.41%). TOST analysis revealed that the *D*_W_ values of the water-filled funnel phantom computed with our algorithm are statistically equivalent to actual water-phantom diameters and to manually computed *D*_W_ values slice by slice, as prescribed by the IEC 62985:2019 norm. Bland–Altman analysis corroborates those findings, giving a smaller value for the LoA (<0.1%) than the one the IEC requires.

The processing times (see **Table 2**) are compatible with a clinical workflow. In particular, the maximum time for processing a study is about 1.5 min (99 s) for 4,460 slices. Moreover, 75% (third quartile) of the studies required a processing time below 16 s. The mean time to process a single study is 11.52 s (the mean number of slices is 789).

These results can lead to the building of a real-time *D*_W_ and SSDE and *D*_W_ computation system, allowing users (radiologists, radiographers, and medical physicists mainly) to obtain size-specific dosimetric information in short times after the very first scan of a patient. This should permit checking if the protocol used on the patient is tailored to its build or needs optimization.

Having decoupled the computation from the DICOM storage, we are sure that our architecture does not interfere with the existing DICOM network environment.

The accuracy achieved in the SSDE and *D*_W_ computations and the very small time for processing an entire CT study allow us to compute the water-equivalent diameter averaged over the slice *D*_w_ave_ (17), a quantity favorable for protocol optimization (17). Based on the authors’ knowledge, there are no previous studies showing such small computational times allowing for clinical real-time computation of size-specific dosimetric quantities.

We computed *D*_W_ and corrected it for truncated images, and the data we saved contain the percentage of truncation, so the final user can relate the accuracy of *D*_W_ with the presence of an amount of truncation.

This study has, however, some limitations. First, we computed times without accounting for time latencies in DICOM networks and/or for writing on files. Further studies should investigate these issues, and they should use a database instead of a text file. Second, our software relies massively on the Italian-named CT Protocol Names and thus is not immediately expandable to other clinical realities. Furthermore, we have not tested it on all CT vendors and all the protocols available for CT. Moreover, we applied our algorithm only to CT studies of the head, thorax, and abdomen/pelvis regions. Further studies require testing (or changing/adapting) the algorithm on anatomical districts with multiple body regions, such as extremities, wrist, and shoulders.

## Conclusion

A fully automated method of *D*_W_ measurement in a clinical workflow has been successfully developed. The system shows that it is possible to have highly accurate *D*_W_ and SSDE in almost real time, without affecting the clinical workflow of CT examinations.

## Data availability statement

The datasets presented in this study can be found in online repositories. The names of the repository/repositories and accession number(s) can be found below: https://github.com/massimilianoporzio/computeSSDE.

## Ethics statement

The studies involving human participants were reviewed and approved by Comitato Etico Interaziendale dell’A.O. S. Croce e Carle di Cuneo e delle AA.SS.LL. CN1, CN2 e Asti. Written informed consent for participation was not required for this study in accordance with the national legislation and the institutional requirements.

## Author contributions

MP conceived the study design and developed the software. MP and CA performed data analysis, took part in discussions and preparation of the manuscript, and drafted the manuscript. All authors read, discussed, and approved the final manuscript.

## Funding

This work was funded by the World Class Research University (WCRU), Diponegoro University, No. 118-08/UN7.6.1/PP/2021.

## Acknowledgments

We would like to thank ASL CN1 for the effort in approving of this study in such a brief time.

## Conflict of interest

The authors declare that the research was conducted in the absence of any commercial or financial relationships that could be construed as a potential conflict of interest.

## Publisher’s note

All claims expressed in this article are solely those of the authors and do not necessarily represent those of their affiliated organizations, or those of the publisher, the editors and the reviewers. Any product that may be evaluated in this article, or claim that may be made by its manufacturer, is not guaranteed or endorsed by the publisher.

## Footnotes

1 The first slice may not contain a useful body region.

2 A processing time greater than 1.5 times the interquartile range of all processing times.
